# Cockroaches (*Ectobius vittiventris*) in an Intensive Care Unit, Switzerland^1^

**DOI:** 10.3201/eid1503.071484

**Published:** 2009-03

**Authors:** Ilker Uçkay, Hugo Sax, Sandrine Longet Di Pietro, Hannes Baur, Marie-France Boulch, Christophe Akakpo, Jean-Claude Chevrolet, Didier Pittet

**Affiliations:** University of Geneva Hospitals and Faculty of Medicine, Geneva, Switzerland (I. Uçkay, H. Sax, S. Longet-Di Pietro, M.-F. Boulc’h, C. Akakpo, J.-C. Chevrolet, D. Pittet); Natural History Museum, Bern, Switzerland (H. Baur)

**Keywords:** Infestation, cockroach, intensive care unit, infection control, Ectobius vittiventris, Switzerland, letter

**To the Editor:** *Ectobius vittiventris* (Costa) is a field-dwelling cockroach and 1 of 4,000 cockroach species worldwide (*1*). We describe a cockroach infestation of an intensive care unit (ICU). Successful management required knowledge of the ecology of cockroaches and highlighted the need for species-level identification to tailor control strategies.

The University of Geneva Hospitals are a 2,200-bed tertiary healthcare center. The 18-bed medical ICU is located on the ground floor next to an outdoor recreational area and admits ≈1,400 patients/year. Smoking inside hospital buildings by patients and healthcare workers (HCWs) is strictly prohibited. On August 25, 2006, ≈30 cockroaches were observed in the ICU hiding inside oxygen masks, moving around on the light panels below the ceilings, or dropping onto intubated patients during the night.

An outbreak investigation was initiated. All work areas, including sinks and material stock areas, were thoroughly searched for cockroaches. External pest control experts identified only 1 species, *E*. *vittiventris*, which had presumably entered the ICU through windows facing the outdoor recreational area. The investigation showed that despite verbal recommendations and being repeatedly forbidden to do so, HCWs had opened the windows secretly with screwdrivers so that they could smoke during night shifts. The infestation was halted within 3 days after information regarding the infestation was provided to HCWs and all windows were bolted shut. In contrast to measures required to deal with a reported infestation in a neonatal ICU (*2*), no other measures such as use of insecticides, review of the air circulation system, or changes in architectural structures were necessary to stop the infestation reported here.

Cockroaches can cause 2 potentially serious health problems. First, they may provoke allergic reactions (*3*). Second, they have been suggested as possible vectors of multidrug-resistant pathogens. In particular, cockroaches that live and breed in hospitals have higher bacterial loads than cockroaches in the community (*4*–*6*). Up to 98% of “nosocomial” cockroaches may carry medically important microorganisms on their external surfaces or in their alimentary tracts (*4*–*9*) and may disseminate these microorganisms by fecal–oral transmission.

Cockroaches are capable of harboring *Escherichia coli* (*6*,*7*), *Enterobacter* spp. (*6*,*8*,*9*), *Klebsiella* spp. (*6*,*7*,*9*), *Pseudomonas aeruginosa* (*6*,*9*), *Acinetobacter baumannii* (*2*), other nonfermentative bacteria (*7*,*9*), *Serratia marcescens* (*7*,*9*), *Shigella* spp. (*6*), *Staphylococcus aureus* (*6*,*7*), group A streptococci (*6*,*7*,*9*), *Enterococcus* spp. (*6*,*7*), *Bacillus* spp. (*7*), various fungi (*6*–*8*), and parasites and their cysts (*6*). An outbreak of extended-spectrum β-lactamase–producing *Klebsiella pneumoniae* in a neonatal unit was attributed to cockroaches (*2*). Pulsed-field gel electrophoresis did not distinguish organisms from the insects from those colonizing infants or causing clinical disease (*2*). Unlike other investigators, we did not cultivate the cockroaches (*6*,*9*).

*E*. *vittiventris* cockroaches are easily confused with *Blattella germanica* (Linnaeus) (the German or croton cockroach), which is probably the most important cockroach pest worldwide (*1*,*9*). In contrast to *B*. *germanica* (*6*,*9*) and other species (Technical Appendix ), *E*. *vittiventris* cockroaches are considered to be harmless and have not been associated with human disease or transmission of pathogens. We did not observe any allergic reactions or an increase in colonization or infection rates of multidrug-resistant organisms. *B*. *germanica* cockroaches are nocturnal, cannot fly, are always encountered within human habitations, and require specialized measures for eradication (*10*).

*E*. *vittiventris* cockroaches live in outdoor areas, do not avoid light, and are active during daytime. Buildings are not a natural habitat. In summer, adult insects can fly inside at night, but because these cockroaches are unable to reproduce inside buildings (*1*), stopping entry from outside halts the infestation. Entry can be stopped by closing windows or using mosquito nets. There is no existing insecticide for eradication of *E*. *vittiventris* cockroaches (*10*), and even if there were, it would not be effective because insects from untreated areas outside would enter continuously (*1*).

*E*. *vittiventris* cockroaches have been recently discovered in Geneva (*10*) and have become the most frequently encountered cockroaches in urban areas of Switzerland for several years (*1*). The reason for this finding remains unknown. The summer of 2003 was remarkably hot and dry in central Europe, thus representing a subtropical climate that usually favors the growth and development of cockroach populations (*1*,*7*). If this warming trend persists, populations of *E*. *vittiventris* cockroaches may continue to expand and similar infestations may occur.

In conclusion, effective control strategies for cockroach infestations depend on identification of cockroach species. In this report, permanent closure of all windows was sufficient to stop the infestation. However, to ensure compliance, it was critical to discuss the purposes of the intervention with HCWs.

## Supplementary Material

Technical Appendix Cockroaches (Ectobius vittiventris) in an Intensive Care Unit, Switzerland
