# Supplementary material for: Cockroaches (Ectobius vittiventris) in an Intensive Care Unit, Switzerland
Source: Emerg Infect Dis. 2009 Mar;15(3):496–7. doi: 10.3201/eid1503.071484 (PMC2666288; doi:10.3201/eid1503.071484)
Supplement: Technical Appendix — Cockroaches (Ectobius vittiventris) in an Intensive Care Unit, Switzerland [file 07-1484_Techapp-s1.pdf]

Cockroaches (*Ectobius vittiventris*) in an Intensive Care Unit, Switzerland

Technical Appendix

Technical Appendix Table. Characteristics of the most common cockroach species*

Scientific name	*Blatta orientalis*	*Blattella germanica*	*Periplaneta americana*	*Ectobius vittiventris*
English name	Oriental cockroach	German cockroach	American cockroach	Field-dwelling cockroach
Habitat	Dark and moist places, damp areas, kitchens. Worldwide.	Domestic. Worldwide.	Subtropical areas worldwide, USA. May survive in dry areas.	Green areas surrounding buildings. Mostly Europe.
Image	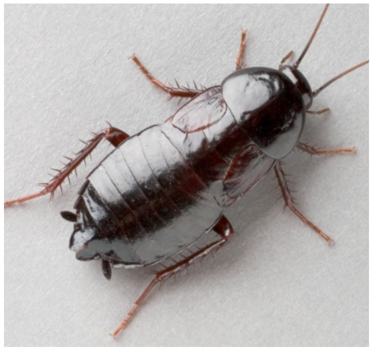	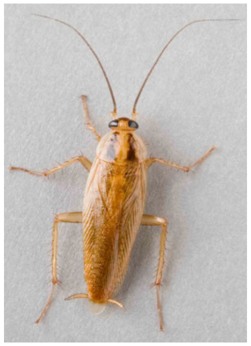	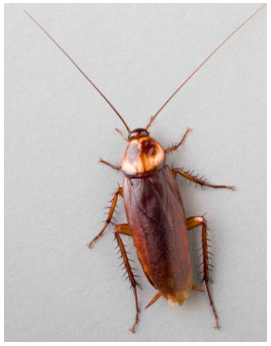	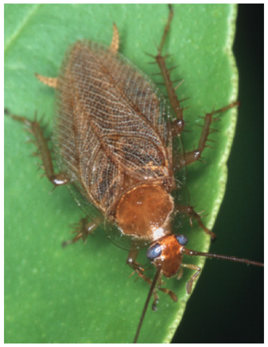
Size, mm	20–30	10–15	35–40	10
Ability to fly	Short distance	No	Yes	Yes
Lifetime, mo	18	18	8–15	≥24
Nuisance	Rarely transmits pathogens	Can transmit pathogens	Can cause allergic disease and transmit pathogens	No
Eradication	Insecticide, e.g., lambda-cyhalothrin	Insecticide, e.g., permethrin, hydromethylnon	Insecticide, e.g., permethrin, hydromethylnon	Keep outside

*Source: Swiss Society of Pest Control (www.fsd-vss.ch) and various references. Photographs were obtained from Hannes Baur, Natural History Museum, Bern, Switzerland.
